# Levelling up: Global examples of reducing health inequalities

**DOI:** 10.1177/14034948211022428

**Published:** 2021-06-19

**Authors:** Clare Bambra

**Affiliations:** Population Health Sciences Institute, Faculty of Medical Sciences, Newcastle University, UK

**Keywords:** Social determinants of health, health equity, health disparities, socio-economic, policy, welfare, health care

## Abstract

There are significant inequalities in health by socio-economic status, race/ethnicity, gender, neighbourhood deprivation and other axes of social inequality. Reducing these health inequalities and improving health equity is arguably the ‘holy grail’ of public health. This article engages with this quest by presenting and analysing historical examples of when sizeable population-level reductions in health inequalities have been achieved. Five global examples are presented ranging from the 1950s to the 2000s: the Nordic social democratic welfare states from the 1950s to the 1970s; the Civil Rights Acts and War on Poverty in 1960s USA; democratisation in Brazil in the 1980s; German reunification in the 1990s; and the English health inequalities strategy in the 2000s. Welfare state expansion, improved health care access, and enhanced political incorporation are identified as three commonly held ‘levellers’ whereby health inequalities can be reduced – at scale. The article concludes by arguing that ‘levelling up’ population health through reducing health inequalities requires the long-term enactment of macro-level policies that aggressively target the social determinants of health.

## Introduction

There are significant social inequalities in health by income, race, gender, neighbourhood deprivation and other axes of social inequality [1]. Social inequalities in health are defined in this article as ‘systematic differences in health between different [social] groups within a society. As they are socially produced, they are potentially avoidable and are widely considered unacceptable in a civilised society’ [2]. Across the social gradient, people in less advantaged social groups tend to have worse health outcomes (e.g. the life expectancy in the most deprived neighbourhoods in England is 7–9 years less than in the most affluent [1]). Health inequalities have been increasing in recent decades in many countries [3], reflected in stalling or declining life expectancies in the most disadvantaged communities [4]. The COVID-19 crisis has also exacerbated these trends [5]. Reducing health inequalities has been described as the ‘holy grail’ of public health [6]. However, we are clearly failing to reduce them currently and urgent questions remain about how we can do so in the future.

This article engages with this empirical uncertainty by looking across time and space to identify successful global examples of when health inequalities have actually been reduced and identifying commonalities and lessons to be learned. In his authoritative account of the history of economic inequality, Walter Scheidel [7] uses multiple examples from the Stone Age to the 20th century to identify four ‘great levellers’ of inequality: mass-mobilisation warfare (e.g. the First and Second World Wars); transformative revolutions (e.g. the communist revolutions in Russia and China and the French Revolution); state collapse (e.g. the collapse of the Tang dynasty in China and the Roman Empire); and global pandemics (e.g. the Black Death in 14^th^-century Europe and the small pox, measles and typhus epidemics in the 16th century Americas).

Following Scheidel [7], this essay retrospectively examines five historical post-war examples from across the world where we have successfully achieved population-level reductions in health inequalities at national and regional scales: the Nordic social democratic welfare states from the 1950s to the 1970s; the Great Society programmes of the 1960s in the USA; democratisation in Brazil in the 1980s; German reunification in the 1990s; and the English health inequalities strategy in the 2000s. The discussion identifies three common ‘levelling’ mechanisms for reducing health inequalities that span the different global examples: welfare state expansion, improved health care access, and enhanced political incorporation. Together they provide useful lessons for future public health action on ‘levelling up’ population health.

## Levelling up: Five global examples

### Example 1 – The social democratic welfare states: Nordic countries, 1950s–1970s

After the Second World War, welfare states were established in most European countries leading to significant improvements in public housing, health care, and the other main social determinants of health including workers enjoying the highest share of national income ever [8]. Post-war welfare states varied and the most encompassing were established in the Nordic countries (Denmark, Finland, Norway and Sweden) [9]. Their social democratic approach was characterised by universal and comparatively generous benefits, collectivism, solidarity (incorporating the working class and the middle classes), a commitment to full employment and income protection and a strongly interventionist state [10]. The state was used to promote equality through pre-taxation wage compression organised via strong collective bargaining and the incorporation of the trade union movement within the state; and by using the taxation system to redistribute via the welfare state social security system [10]. This meant that from the 1950s/60s to the 1980s income inequalities were the smallest – and poverty rates the lowest – in these countries [11].

This led to lower (absolute) health inequalities in these countries. The 1980 British Black Report contained a range of comparative data from the 1970s about health inequalities across Europe. It showed that Norway and Sweden had the smallest (and reducing) socio-economic inequalities in mortality, particularly in comparison to France, West Germany and the UK [12]. Other comparative studies of mortality conducted in the 1970s and 1980s came to similar conclusions. For example, a study by Valkonen examined educational inequalities in mortality in six European countries in the 1970s. It found that relative inequalities were largest in France, then the UK and Finland whilst they were smallest in Denmark, Norway and Sweden [13]. This was reinforced by subsequent studies of morbidity that compared Sweden and the UK [14,15]. In this period, the Nordic countries also had the lowest mortality rates across all social classes [16].

The ‘golden age’ of the welfare state effectively ended with the economic crisis of the 1970s (high inflation, slow economic growth, the end of full employment) [17] and the emergence of neoliberal economics (i.e. ‘market fundamentalism’) – initially in the Anglo-American countries but then spreading across continental Europe in the 1980s and 1990s [18]. Neoliberalism led to the erosion of the post-war social democratic welfare model and an increase in income (and health) inequalities [18].

### Example 2 – The Great Society, civil Rights and the war on poverty: 1960s USA

In the USA in the 1960s President Lyndon B. Johnson announced the ‘Great Society’ policy programme that led to a series of substantial programmes to address inequalities in medical care, civil rights, education and poverty [19]. The Medicare (1965: universal health insurance for all over 65s) and Medicaid (1966: limited health care costs coverage for welfare recipients) programmes were introduced [20]. These substantially increased access to health care for the poorest groups [20]. The ‘war on poverty’ included various initiatives to address urban and rural poverty: increased educational opportunities (including significant increases in federal funding for the education system); expansion of the federal food stamp programme; increased state pension value; and expansion of the scope of the main federal welfare programme – Aid for Dependent Children – to cover black mothers [21]. The 1964 Civil Rights Act outlawed racial discrimination (which led to the abolition of the legal system of racial discrimination in the 21 southern states and District of Columbia, called ‘Jim Crow’) [22] leading to the desegregation of schools and public accommodations (including hospitals), and equalised voting rights [23].

A series of analyses by Krieger and colleagues has examined the impact of these reforms on health inequalities [24–27]. They found that racial and income inequalities in premature mortality (deaths under the age of 75) and infant mortality rates (IMRs; deaths before the age of 1) declined between 1966 and 1980 after the war on poverty and the enactment of civil rights legislation [24]. Their analysis of trends in inequalities in breast cancer rates between black (non-Hispanic) and white American women (1992–2012) in former Jim Crow states compared with non-Jim Crow states found that there was a significantly greater level of racial inequality in Jim Crow compared with non-Jim Crow states amongst women born before 1965 (born before 1945: odds ratio 1.09 [95% CI 1.03, 1.14]; born 1946–1965, 1.06 [1.01, 1.11]) [25]. These between-state differences disappeared amongst women born after the abolition of Jim Crow in 1965 (1.02 [0.90, 1.17]) [25]. The positive impact of Jim Crow abolition has also been demonstrated with regard to racial inequalities in premature mortality and IMRs [26,27].

Health inequalities in the USA then increased again between 1980 and 2002 during the Reagan–Bush period [24] of neoliberalism when public welfare services (including health care insurance coverage) were cut, funding of social assistance was reduced, the minimum wage was frozen and the tax base was shifted from the rich to the poor leading to increased income polarisation [2,18].

### Example 3 – Democratisation: Brazil 1980s to 2000s

In 1985, Brazil started a gradual transition from military dictatorship (1964–1985) to become a stable democracy by the mid-2000s. This increased political participation was accompanied by an expansion of health and welfare programmes, including the introduction of universal health care in 1988 (the Unified Health System); a national women’s health programme and a national programme for child health in 1984; a family health programme in 1994; a national programme for the reduction of infant mortality in 1995; and the Bolsa Família cash transfer programme for low-income women with children in 2003. These led to a significant improvement in maternal and child health care and a reduction in Brazil’s poverty rates as well as a decrease in income inequalities between the rich and poor [28–30].

Since these reforms, the IMR in Brazil has fallen by more than 70%: from 83 per 1000 live births in 1980 to 47 per 1000 live births in 1990, to 13.3 in 2015 [29]. This is one of the fastest drops in infant mortality ever recorded worldwide and higher than would be expected by the increase in Brazil’s gross domestic product per capita [30]. Regional differences in IMR and differences between rich and poor social groups also decreased [29]. For example, the difference in mortality rates between the top and bottom wealth quintiles decreased from 65 deaths per 1000 children in 1991 to 31 deaths per 1000 in 2001–2002 [30]. Similarly, other indicators of child health inequalities improved: in 1989, children from families in the lowest wealth quintile were 7.7 times more likely to have stunted growth than those from families in the highest wealth quintile [31]. This ratio stabilised at around 6.6 in 1998 and reduced sharply to 2.6 in 2007 [31].

However, Brazil’s reductions in health inequalities and improvements in population health are under threat from the economic and political crises in the country since 2015. Brazil experienced a significant economic recession in 2015 which was followed by the implementation austerity measures including a substantial reduction in expenditure on – and population coverage of - social welfare programmes - including Bolsa Família - potentially increasing poverty rates. Democracy has also in Brazil declined since the election of President Jair Bolsonaro in the 2018 election and the country is also suffering from very high rates of COVID-19.

### Example 4 – Fall of communism and reunification: Germany in the 1990s

In 1990, the life expectancy gap between the former East and the former West of Germany was almost three years for women and three and a half years for men. This gap rapidly narrowed in the following decades so that by 2010 it had dwindled to just a few months for women (West: 82.8 years; East: 82.6 years) and just over one year for men (West: 78.0 years, East: 76.6 years) [2]. This provides an important example of how health inequalities can be reduced – significantly, at scale and in a fairly short time frame. How was this done?

Firstly, the living standards of East Germans improved with increases in wage levels and better access to a variety of foods and consumer goods [32]. This particularly benefitted old age pensioners in the East as the West German pension system was extended into the East, which resulted in huge increases in income for older East Germans [32]. Research by the Max Planck Institute for Demographic Research in Rostock has shown that the rapid improvement in life expectancy in 1990s East Germany was largely a result of falling death rates amongst pensioners [32].

Secondly, immediately after reunification, considerable financial support was given to modernise the hospitals and health care equipment in the East, and the availability of nursing care, screening and pharmaceuticals also increased. This raised standards of health care in the East so that they were comparable to those of the West within just a few years [33]. This had notable impacts on, for example, improvements in neonatal mortality in the East and in mortality from conditions amenable to prevention (e.g. cancer screening) or medical treatment [34].

Both the improvement in living standards and the increased investment in health care were the result of the deep and sustained *political* decision to reunify Germany as fully as possible so that – in the words of German Chancellor Helmut Kohl (1982–1998) – ‘what belongs together will grow together*’* [2]. Indeed, the improvements in the East were funded by a special Solidarity Surcharge: an additional income tax charge paid across Germany [2].

### Example 5 – National health inequalities strategy: England in the 2000s

In 1997, a Labour government (social democratic) was elected in the UK on a manifesto that included a commitment to reducing health inequalities. This led to the implementation, between 2000 and 2010, of a wide-ranging and multi-faceted health inequalities reduction strategy for England in which policymakers systematically and explicitly attempted to reduce inequalities in health [2]. The strategy focused specifically on supporting families, engaging communities in tackling deprivation, improving prevention, increasing access to health care and tackling the underlying social determinants of health. For example, the strategy included large increases in levels of public spending on a range of social programmes, the introduction of the national minimum wage, area-based interventions such as the Health Action Zones, and a substantial increase in expenditure on the health care system [35]. What was the impact of this effort on health inequalities?

These policies led to reductions in social inequalities in the key social determinants of health, including unemployment, child poverty, housing quality, access to health care, and educational attainment [2]. These were accompanied by modest reductions in health inequalities between the most deprived areas in England and the rest of the country [36–38]: inequalities in life expectancy decreased by just over a year for men and around six months for women [36]; the gap in IMRs narrowed by 12 infant deaths per 100,000 births per year [37]; and inequalities in mortality amendable to health care interventions decreased by 35 deaths per 100,000 for men and 16 deaths per 100,000 for women [38].

The English strategy of the 2000s therefore reduced health inequalities but the decreases were on the modest side. Arguably, it may have been even more effective if there had not been a gradual ‘lifestyle drift’ in governance, whereby policy went from thinking about the social determinants of health to focusing almost exclusively on individual-level behaviour change [35]. The strategy also did not significantly address the more fundamental social and economic causes of inequality [35] and was highly medicalised in its framing of inequalities [39]. The strategy may also have been even more effective if it had been sustained over a longer time period, but from 2010 the newly elected Conservative–Liberal coalition government pursued a policy of austerity, which has been associated with increasing health inequalities [40].

## Discussion: The three ‘levellers’ of health inequality

Scheidel [7] used his historical examples to identify four great levellers of economic inequality: mass-mobilisation warfare, transformative revolutions, state collapse, and global pandemics. Similarly, it is possible to identify common levellers across these five examples of when health inequalities were reduced at scale, namely welfare state expansion, improved health care access, and enhanced political incorporation. Common to all five examples is the expansion of social security safety nets (and the reduction of poverty) and increased health care access particularly for the poorest groups. Likewise, Examples 1, 2, 3 and 4 are characterised by the political incorporation of the working classes (via corporatism in Case Study 1, democratisation in Case Studies 3 and 4) and/or minority groups (black Americans in Example 2 and democratisation in Example 3).

Further evidence of the importance of these three mechanisms for reducing health inequalities comes from studies of reductions in social security and health care provision. For example, Krieger et al.’s analysis of time trends in inequalities in IMR and premature mortality in the USA found that whilst inequalities decreased during a period of welfare state expansion (Example 2), they increased again when social security was reduced under Reagan in the 1980s [24]. Similar associations have been found between the expansion and contraction of the welfare state and post-war trends in health inequalities in the UK and New Zealand [41,42]. More recent research into the impact of austerity policies (cuts to health care and welfare budgets) in Europe has also found that health inequalities increased [43,44]. For example, as child poverty decreased between 2000 and 2010 in England (Example 5), inequalities in IMR decreased. However, as child poverty rates increased between 2010 and 2020 – the decade of austerity – inequalities in IMR increased again [40]. This ‘dose–response’ relationship between social security provision and health inequalities was also identified in a recent systematic review of 38 studies [45]. There is therefore an association between the ‘waxing and waning’ of the welfare state and health inequalities: as welfare state provision increases (and poverty decreases), health inequalities fall; when the welfare state is reduced, health inequalities tend to increase.

These three mechanisms are not independent of one another though – historically, democratisation and the political incorporation of the working classes and minority groups has tended to result in increases in welfare state and health care provision [10]. It also needs to be acknowledged that these mechanisms are not universally effective. For example, political incorporation has not always led to social policy and health care improvements or reduced health inequalities (e.g. the dismantling of apartheid in South Africa post-1990 was not associated with reductions in inequalities in infant mortality) [46]. Likewise, research into the Scandinavian ‘public health puzzle’, whereby, despite extensive welfare states, universal health care and high levels of political participation, Scandinavian countries have not eradicated health inequalities (potentially as a result of inequalities in access to health care and social welfare, educational opportunities or health behaviours) [47–49]. Therefore, the details of policy context and implementation appear to matter for these mechanisms to be effective and this needs to be explored further – through empirical work – especially in relation to ascertaining causality and intermediate mediators (including the role of local-level – as well as macro-level – policy actions). Indeed, this commentary only sets out an argument – albeit supported by analysis of historical examples. Further research using empirical methods is needed to explore the mechanisms identified; systematic review methods may be particularly beneficial in this regard [46].

This essay has identified empirical examples of instances when population-level health inequalities have reduced and, in examining them together, has enabled common mechanisms to be identified. The analysis has also highlighted the need for policy action to be sustained over long periods (the five examples all span at least a decade) and for there to be sufficient political will to sustain it [2]. It also suggests that globally, health inequalities research needs to move on from trying to identify the single, ‘silver-bullet’ intervention that will reduce health inequalities, and focus more on the implementation and evaluation of wide-ranging, long-term policy programmes that simultaneously target multiple social determinants of health [50]. This will help us to develop more effective post-pandemic public health policy programmes.

## Conclusion

Together, the five global examples presented in this essay suggest that there are three common levelling mechanisms whereby social gradients in health can be reduced at scale: by improving social and economic conditions through more expansive social policies, increasing health care access, and through democratisation and political incorporation. The public health community needs to learn from these past experiences – quickly – to prevent inequality growing post-COVID-19 pandemic and to reduce health inequalities in the future.
